# Bariatric surgery: behavioral patterns and personality disorders in the preoperative period

**DOI:** 10.15649/cuidarte.2921

**Published:** 2024-05-20

**Authors:** Michelle Diniz da Mata, Samuel Barroso Rodrigues, Camila Souza de Almeida, Sebastiáo Júnior Henrique Duarte, Ricardo Bezerra Cavalcante, Richardson Miranda Machado

**Affiliations:** 1 Universidade Federal de Sáo Joáo del-Rei. Divinópolis, Brasil. michelledmpsicologa@gmail.com Universidade Federal de São João del-Rei Universidade Federal de Sáo Joáo del-Rei Divinópolis Brazil michelledmpsicologa@gmail.com; 2 Universidade de Itaúna. Itaúna, Brasil. samuelbarroso88@gmail.com Universidade de Itaúna Universidade de Itaúna Itaúna Brazil samuelbarroso88@gmail.com; 3 Universidade do Estado de Minas Gerais. Divinópolis, Brasil. camila.almeida@uemg.br Universidade do Estado de Minas Gerais Universidade do Estado de Minas Gerais Divinópolis Brazil camila.almeida@uemg.br; 4 Universidade Federal do Mato Grosso do Sul. Campo Grande, Brasil. sjhd.ufms@gmail.com Universidade Federal do Mato Grosso do Sul Universidade Federal do Mato Grosso do Sul Campo Grande Brazil sjhd.ufms@gmail.com; 5 Universidade Federal de Juiz de Fora. Juiz de Fora, Brasil. ricardocavalcante.ufjf@gmail.com Universidade Federal de Juiz de Fora Universidade Federal de Juiz de Fora Juiz de Fora Brazil ricardocavalcante.ufjf@gmail.com; 6 Universidade Federal de Sáo Joáo del-Rei. Divinópolis, Brasil. richardson@ufsj.edu.br Universidade Federal de São João del-Rei Universidade Federal de Sáo Joáo del-Rei Divinópolis Brazil richardson@ufsj.edu.br

**Keywords:** Psychiatric Nursing, Bariatric Surgery, Personality Disorders, Behavior, Enfermería Psiquiátrica, Cirugía Bariátrica, Trastornos de la Personalidad, Comportamiento, Enfermagem Psiquiátrica, Cirurgia Bariátrica, Transtornos da Personalidade, Comportamento

## Abstract

**Introduction:**

Bariatric surgery has become an increasingly common procedure, especially for patients with morbid obesity who have obtained unsatisfactory results from conventional treatments.

**Objective:**

To evaluate the occurrence of behavioral patterns and personality disorders in patients in the preoperative period of bariatric surgery.

**Materials and methods:**

Cross-sectional study carried out with 146 patients from a medium-sized clinic, a reference in the execution of bariatric surgeries in the Midwest region of Minas Gerais, Brazil. Data collection was performed using the psychological instrument entitled Factorial Personality Battery. Descriptive analysis and data association were performed.

**Results:**

Half of the participants presented high or very high scores for greater propensity to develop depression and anxiety, showing a close relationship with personality disorders, especially with behavioral patterns of effort and dedication.

**Discussion:**

The patterns of effort and dedication behavior are protective factors in the postoperative period, taking into account the adaptations and new habits necessary for a good recovery and maintenance of weight loss.

**Conclusions:**

The dysfunctional patterns of behavior that stood out most are related to greater difficulty in perceiving the positive side and ease in perceiving the negative side, leading to a more intense experience of suffering, in addition to difficulty in making decisions and facing routine challenges. Screening behavioral patterns and personality disorders preoperatively is necessary for adequate patient monitoring and successful bariatric surgery.

## Resumen

**Introducción:**

La cirugía bariátrica se está convirtiendo en un procedimiento cada vez más frecuente, especialmente en pacientes con obesidad mórbida que han obtenido resultados insatisfactorios con los tratamientos convencionales.

**Objetivo:**

Evaluar la aparición de patrones de conducta y trastornos de la personalidad en pacientes en el preoperatorio de cirugía bariátrica.

**Materiales y métodos:**

Estudio transversal realizado con 146 pacientes de una clínica de mediano tamaño, referencia en la realización de cirugías bariátricas en la región Centro-Oeste de Minas Gerais, Brasil. La recolección de datos se realizó mediante el instrumento psicológico denominado Batería de Factores de Personalidad. Se realizó análisis descriptivo y de asociación de datos.

**Resultados:**

La mitad de los participantes presentaron puntuaciones altas o muy altas de mayor propensión a desarrollar depresión y ansiedad, mostrando una estrecha relación con los trastornos de la personalidad, especialmente con patrones de conducta de esfuerzo y dedicación.

**Discusión:**

Los patrones de conducta de esfuerzo y dedicación son factores protectores posquirúrgicos, teniendo en cuenta las adaptaciones y nuevos hábitos necesarios para una buena recuperación y mantenimiento de la pérdida de peso.

**Conclusiones:**

Los patrones de conducta disfuncionales que más destacaron se relacionan con una mayor dificultad para percibir lo positivo que lo negativo, lo que lleva a una experiencia de sufrimiento más intensa, además de dificultad para tomar decisiones y enfrentar desafíos rutinarios. El seguimiento de los patrones de conducta y los trastornos de la personalidad antes de la operación es necesario para una monitorización adecuada del paciente y una cirugía bariátrica exitosa.

## Resumo

**Introdução:**

A cirurgia bariátrica torna-se um procedimento cada vez mais frequente, principalmente para pacientes com obesidade mórbida que obtiveram resultados insatisfatórios em tratamentos convencionais.

**Objetivo:**

Avaliar a ocorréncia de padroes comportamentais e de transtornos da personalidade em pacientes no pré-operatório da cirurgia bariátrica.

**Materiais e métodos:**

Estudo transversal realizado com 146 pacientes de uma clínica de médio porte, referencia na execugáo de cirurgias bariátricas na regiáo Centro-Oeste de Minas Gerais, Brasil. A coleta de dados foi realizada por meio do instrumento psicológico intitulado Bateria Fatorial de Personalidade. Realizou-se análise descritiva e de associagáo dos dados.

**Resultados:**

Metade dos participantes apresentou escores altos ou muito altos para maior propensáo ao desenvolvimento de quadros de depressáo e ansiedade, apresentando estreita relagáo com os transtornos da personalidade, sobretudo com os padroes de comportamento de esforgo e dedicagáo.

**Discussáo:**

Os padroes de comportamento de esforgo e dedicagáo sáo fatores protetivos no pós-cirúrgico, levando-se em conta as adaptagoes e novos hábitos necessários para uma boa recuperagáo e manutengáo da perda de peso.

**Conclusões:**

Os padroes disfuncionais de comportamento que mais se destacaram estáo relacionados a maior dificuldade na percepgáo do positivo em detrimento do negativo, levando a vivencia mais intensa de sofrimento, além da dificuldade na tomada de decisoes e no enfrentamento de desafios rotineiros. O rastreamento de padroes comportamentais e transtornos da personalidade no pré-operatório torna-se necessário para o adequado acompanhamento dos pacientes e proveniente sucesso da cirurgia bariátrica.

## Introduction

Obesity is a disease with vast growth worldwide, affecting children, adolescents and adults. Being a major public health problem, it reaches 30% of the population. Data indicate a total of 379 million overweight or obese children and 1.9 billion overweight adults in the world^1^.

In Brazil, there was an increase in the prevalence of obesity in adults from 11.8% in 2006 to 20.3% in 2019. The increase occurred mainly in the population aged 18 to 24 years, with a higher level of education, according to a study by Silva et al.^2^ in all capitals of the country. In addition, there is evidence of an increase in the prevalence of morbid obesity in the adult population in Brazilian capitals between 2006 and 2017. The rates in this period vary between 1.3% and 1.9% in the female population and between 0.9% and 1.3% in the male population. The highest growth is presented in the age group from 25 to 44 years, from 0.9% to 2.1%^3^.

Thus, bariatric surgery is a procedure that has become increasingly frequent, especially for patients with morbid obesity who have obtained unsatisfactory results in conventional treatments. Francisco et al.^4^ state that 95% of these patients regain their weight after two years in a traditional clinical approach. However, the World Health Organization^1^ expects some results such as weight loss, improvement of related comorbidities and quality of life in patients undergoing the procedure but reinforces the need for attention to psychiatric disorders that may be pre-existing or arise after the intervention.

Bordignon et al.^5^ point to the significant prevalence of psychiatric disorders in candidates for bariatric surgery, including personality disorders. Similar results were found by Giulietti et al.^6^, who emphasize the importance of investigating these diseases for early intervention and adequate monitoring of these patients.

Personality can be conceptualized as a pattern of behavior, emotions and thoughts of the individual. In the conformation of this pattern, there are personality traits that will configure how the individual will express and live his/her emotions, feelings, interpersonal behaviors, loving and spiritual experiences, in addition to ideological, community and criminal expressions. Personality traits dialogue with ethical and moral principles of the society in which the individual developed^7^.

When there are persistent patterns of emotions, thoughts and behaviors, which lead the individuals to maladaptive responses to their environment, causing damage and suffering, there are personality disorders that are serious psychiatric dysfunctions and affect about 10% of the population and cause high health costs^7^^,^^8^.

It is important to highlight that changes in behavior patterns and personality disorders can impair preoperative preparation for the procedure and full postoperative recovery, contributing to undesirable results. Thus, adequate screening for the detection of personality disorders and preoperative behavior patterns becomes essential for the promotion of patients' mental health and the success of the intervention. 

This research evaluated the occurrence of behavioral patterns and personality disorders in patients in the preoperative period of bariatric surgery. Adequate screening, which is part of the nurse's duties, is crucial in detecting personality disorders and behavior patterns in the preoperative period, as they can harm the preparation for the procedure in the preoperative period and full recovery in the postoperative period. The scarcity of studies in the literature on this topic further reinforces the importance of this study, especially so that there is guidance on the promotion of mental health and nursing care for patients with personality disorders under these conditions. 

Therefore, this study aims to evaluate the occurrence of behavioral patterns and personality disorders in patients in the preoperative period of bariatric surgery.

## Materials and Methods

This is a cross-sectional, exploratory-analytical study with a quantitative approach. The study was developed in a medium-sized clinic, a reference in the execution of bariatric surgeries for the 56 municipalities in the Midwest region of Minas Gerais, Brazil from supplementary health care.

The population of this study was composed of patients treated at a metabolic obesity surgery clinic, which offers various services and has a multidisciplinary team, including nursing, psychology, nutrition, surgery, clinic, trichology, ultrasound and endoscopy.

The sample calculation was based on the number of bariatric surgeries performed by the Clinic from 2001 to 2020, totaling 1,416 patients. Thus, a projection of the growth in the number of surgeries in the last 19 years was made. The increment and its pattern over time were observed via scatter plot. Thus, there was a linear growth in the number of surgeries over the 19 years, which made it possible to adjust a model to predict the number of surgeries for 2020 as follows: total number of surgeries "1,416" divided by 19 = mean 74.5 surgeries per year. From the analysis of the growth in the number of surgeries year by year, a mean increase in the number of surgeries of 10.5% per year was identified. Thus, the number of surgeries expected for 2020 was 148. Thus, for the sample calculation, a proportion of 50% was considered for a given characteristic (because it is a project with multiple outcomes), a value that provided the largest sample size (93), setting the level of significance at 5% (alpha or type I error) and the sampling error at 5%. Data collection was carried out from January 2020 to December 2020. All 149 patients who would undergo bariatric surgery were invited to participate in the research, according to the schedule provided by the aforementioned clinic. Preoperatively, 146 patients were evaluated, almost the entire population that would undergo bariatric surgery. Only 3 patients did not participate because they gave up on the operation.

The following inclusion criteria were established: age equal to or greater than eighteen years and literate. Exclusion criteria: difficulty in reading and interpreting and/or incomplete information in completing the response protocol.

The Factorial Personality Battery (FPB) was used for data collection. This is a psychological instrument constructed to assess personality and behavior patterns based on the "Big Five Factors" model.

The instrument consists of 126 items that cover the feelings, opinions and attitudes of the individual. The items evaluated include the five personality factors: Neuroticism, Socialization, Achievement, and Openness to experience and Extroversion. The answers are indicated on a seven-point Likert scale according to the degree of identification of the individual with each sentence presented^9^.

The test allows the identification of behavioral tendencies and more likely patterns of attitudes and beliefs, but in no situation can it be said that "certainly" the individual for having a characteristic will behave in such a way or not. After calculating the scores, the percentile points are verified and the scores are classified as very low, low, medium, high and very high. Extreme scores may indicate a maladaptive personality pattern^9^. The test was approved for use in Brazil, after a study with a sample of 6,599 individuals^9^.

The instrument was applied collectively, with a mean duration of 40 minutes and complied with all protocols in force for COVID-19.

After the application of the Factorial Personality Battery, the data were released on the "Q-Web Platform", made available by "Pearson Clinical Brasil", which performs the web correction free of charge to professionals who purchase the response protocols from authorized resellers. The platform made available after correction a report of each patient, with scores and percentiles of all factors and facets evaluated, which are presented through tables, graphs and interpretative analysis.

The general analysis of the distribution of each of the Big Five Factors of personality and all its facets was performed by compiling the data in Microsoft Excel through descriptive statistics. Data were cross-referenced, based on the interpretation proposed by the FPB technical manual and on the diagnostic criteria of DSM-V^10^ to identify possible personality disorders. In order to evaluate the association/independence between these variables, Fisher's Exact Test was used, since some levels of the variables under study had a low frequency of occurrence. Qualitative variables were analyzed using Fisher's Exact Test, while the quantitative variable “age” was subjected to the Student’s t-test. The Shapiro-Francia test was performed to determine the normality of the data. The research database can be checked on the Mendeley Platform^11^.

The data collected for this research complied with all the norms and safeguards established by the Research Ethics Committee of the Federal University of Sao Joao Del-Rei, Midwest Dona Lindu Campus (UFSJ/CCO). The research participants were asked to read and sign the Informed Consent Form, therefore, the entire process followed the terms of Resolution number 466, of December 12, 2012, of the National Research Ethics Council, which deals with rules on research involving human beings. The study was approved under opinion number 3,330,917.

## Results

In this study, 146 patients were evaluated in the preoperative period of bariatric surgery. Regarding the characteristics of patients according to sex, there was a predominance of females with 108 (73.97%) patients, with regard to age group, 30 to 39 years old prevailed with 54 (37%) patients, followed by from 40 to 49 years old with 38 (26%) patients. Table 1 below presents the distribution of patients with regard to the Big Five personality factors.

After a detailed analysis of the differences between men and women in relation to the "Big Five Factors", no significant differences were identified, as indicated by the p values > 0.05 obtained in the corresponding statistical tests. These results suggest that, broadly speaking, men and women exhibit similar personality patterns when it comes to these specific traits, highlighting the importance of understanding individuality and diversity within each gender. Statistical tests play a crucial role in corroborating these values by providing an objective basis for the conclusions reached (Table 1).

**Table 1:** Distribution of patients according to the “Big Five Factors” of personality, Divinópolis, MG, Brazil, 2022.

Sex				
Factor	Total (146) %(n)	Women (108) %(n)	Men (38) %(n)	p-Value
Mean age ± SD	36.80 ± 10.81	36.14 ± 10.67	38.68 ± 11.15	0.2150
Neuroticism				0.352
Very high	29.45(43)	32.41(35)	21.05(8)	
High	18.49(27)	20.37(22)	13.16(5)	
Medium	44.52(65)	38.89(42)	60.53(23)	
Low	6.84(10)	7.41(8)	5.26(2)	
Very low	0.68(1)	0.93(1)	0	
Extroversion				0.535
Very high	14.38(21)	12.96(14)	18.42(7)	
High	19.86(29)	20.37(22)	18.42(7)	
Medium	45.89(67)	43.52(47)	52.63(20)	
Low	6.84(10)	8.3(9)	2.63(1)	
Very low	13.01(19)	14.81(16)	7.89(3)	
Socialization				0.428
Very high	8.90(13)	11.11(12)	2.63(1)	
High	13.01(19)	12.96(14)	13.15(5)	
Medium	52.05(76)	5.77(57)	50.00(19)	
Low	18.49(27)	16.66(18)	23.68(9)	
Very low	7.53(11)	6.48(7)	10.52(4)	
Achievement				0.828
Very high	5.47(8)	0.92(1)	18.42(7)	
High	8.90(13)	4.62(5)	21.05(8)	
Medium	22.60(33)	17.59(19)	36.84(14)	
Low	9.58(14)	8.33(9)	13.15(5)	
Very low	5.67(8)	3.70(4)	10.52(4)	
Openness				0.093
Very high	1.36(2)	0.92(1)	2.63(1)	
High	4.79(7)	2.77(3)	10.52(4)	
Medium	31.50(46)	36.11(39)	18.42(7)	
Low	27.39(40)	25(27)	34.21(13)	
Very low	34.93(51)	35.18(38)	34.21(13)	

P-value Qualitative variables were analyzed using Fisher's Exact Test, while the quantitative variable "age" was subjected to the Student's t-test.

P-values were calculated and can be found in Table 2. When analyzing the "Factor Facets", it is observed that no significant differences were identified between men and women, except for the Pro-Sociability of the Socialization factor, where a p value less than 0.05 was found (p = 0.027). In addition, in relation to the "Search for Novelties" of the "Openness" dimension, there was a significant difference, with a p value less than 0.05 (p = 0.007).

**Table 2:** Distribution of patients according to personality facets of each of the “Big Five Factors”. Extreme scores allow you to identify the most likely trends and behavior patterns

Factors x Facets	Women (108)					Men (38)					p-Value
Neuroticism	Very high	High	Medium	Low	Very low	Very high	High	Medium	Low	Very low	
Vulnerability	25.10(27)	21.2(23)	44.30(47)	4.50(6)	4.65(5)	20.65(8)	20.65(8)	44.65(17)	7.67(3)	4.56(2)	0.980
Emotional instability	16.02(17)	8.30(9)	60.01(65)	9.57(11)	4.47(6)	4.56(2)	15.76(6)	46.15(18)	23.87(9)	7.67(3)	0.060
Passivity Lack of Energy	19.05(20)	19.40(20)	54.13(58)	4.47(6)	2.78(4)	23.87(9)	15.76(6)	24.53(22)	0	2.76(1)	0.680
Depression	33.4(36)	28.20(30)	37.32(40)	0.9(1)	0.9(1)	23.87(9)	25.76(10)	44.65(17)	4.56(2)	0	0.502
Extroversion											
Communication Level	9.78(11)	11.54(13)	47.65(52)	19.05(20)	10.76(12)	0	10.65(4)	65.34(25)	20.65(8)	2.76(1)	0.071
Haughtiness	11.54(13)	25.67(28)	34.56(38)	10.76(12)	16.02(17)	25.76(10)	10.65(4)	41.92(16)	10.65(4)	8.93(4)	0.171
Dynamism/Assertiveness	10.76(12)	12.58(14)	47.65(52)	9.78 (11)	17.55(19)	20.65(8)	17.87(7)	44.65(17)	4.56(2)	8.93(4)	0.501
Social Interactions	12.58(14)	17.55(19)	47.65(52)	9.78 (11)	10.76(12)	15.76(6)	17.87(7)	46.15(18)	7.67(3)	8.93(4)	0.992
Socialization											
Kindness	20.75(23)	14.85(16)	50.87(55)	8.75(10)	2.78(4)	20.65(8)	7.83(3)	44.65(17)	17.87(7)	7.67(3)	0.355
Sociability	28.65(31)	8.75(10)	43.72(47)	7.65(8)	10.76(12)	4.56(2)	7.83(3)	39.45(15)	20.65(8)	8.93(4)	0.027
Confidence in People	2,78(4)	7,87(9)	37.58(41)	30.76(34)	19.05(20)	7.67(3)	7.67(3)	14.25(16)	25.76(10)	15.76(6)	0.805
Achievement											
Competence	16.02(17)	16.02(17)	42.93(46)	14.85(16)	10.76(12)	17.87(7)	7.67(3)	52.34(20)	15.76(6)	4.56(2)	0.593
Weighting/Prudence	9.78 (11)	17.64(18)	49.65(54)	8.75(10)	13.87(15)	4.56(2)	10.65(4)	53.54(21)	17.87(7)	8.93(4)	0.602
Engagement/Commitments	20.78(21)	17.64(18)	47.65(52)	8.75(10)	5.65(7)	14.86(5)	23.87(9)	44.65(17)	4.56(2)	14.86(5)	0.490
Openness											
Openness to Ideas	2.78(4)	4.47(6)	35.85(39)	25.10(27)	29.75(32)	2.76(1)	7.67(3)	33.76(13)	15.76(6)	39.45(15)	0.729
Liberalism	1.8(2)	7.65(8)	45.76(50)	21.76(24)	21.86(24)	2.76(1)	7.67(3)	44.65(17)	15.76(6)	28.76(11)	0.836
Search for News	0.9(1)	11.54(13)	50.87(55)	19.76(22)	16.02(17)	14.86(5)	7.67(3)	53.54(21)	4.56(2)	17.87(7)	0.007

Factorial Personality Battery. Source: survey data, 2022. p-Value Fisher's Exact Test.

In the evaluation of the 146 patients, there was a prevalence of the Neuroticism factor. About half of the respondents had high or very high scores for Neuroticism; the female sex was identified as the majority, indicating a more intense experience of emotional suffering, with a tendency to emphasize the negative aspects of the events to the detriment of the positive ones.

As for the bidirectional association, neuroticism showed a significant relationship with obesity, highlighting the behavior patterns related to Depression, Vulnerability and Passivity.

Table 3 below presents the personality disorders found in the research from the crossing of data based on the interpretation proposed by the FPB technical manual and the DSM-V diagnostic criteria.

The research results suggest the presence of some type of personality disorder in 14 patients (11 women and 3 men), which represents 9.5% of the total sample (146), with 3 (three) patients meeting the criteria for paranoid PD, 3(three) for borderline PD, 2 (two) for narcissistic PD, 3 (three) for avoidant PD and 2 (two) for obsessive-compulsive PD.

There was a higher prevalence of PD belonging to groups B, characterized by emotionality and inconstancy, and of PD belonging to group C of the DSM-V, characterized by fear and anxiety.

**Table 3:** Distribution of patients according to the identification of Personality Disorders, based on the "Big Five Factors", Divinópolis, MG, Brazil, 2022

Big Five Factors and Personality Disorders							
Factors/Scores					**Personality Disorders**	Women	Men
Neuroticism	Extroversion	Socialization	Achievement	Openness			
High scores, especially in Emotional Instability	-	Low scores, in particular in confidence in People and Social interaction	-	-	Paranoid	2	1
High Scores	-	Low scores in Confidence in people	Low Scores	-	Borderline	3	0
High Scores	Low Scores		Low Scores in Competence	-	Avoidant	3	0
-	High scores in haughtiness	Low scores on Kindness and Pro-Sociability	-	-	Narcissist	2	1
-	Very high scores in Dynamism Assertiveness	-	High Scores	Low scores, especially in Openness to Ideas and Liberalism	Obsessive Compulsive Disorder	1	1
Total						11	3

* Factorial Personality Battery. Source: Research data, 2022.

## Discussion

The research showed a higher frequency of women who would undergo bariatric surgery, a fact that may be related to the higher prevalence of obesity in females in most countries. It is assumed that the preponderance ofwomen is associated with two main points: greater tendency to seek health care in general and concern with body image. Compared to men, women are more likely to seek all types of obesity treatment, including bariatric surgery^12^^,^^13^.

With regard to age, most patients were aged between 30 and 39 years and between 40 and 49 years. The pre-eminence ofthese age groups may indicate other previous priorities, such as the commitment to work/career and for women we still consider the dedication to motherhood/family. A recent study by Hales et al.^14^ points to a prevalence of obesity of more than 40% among adults aged 20 to 59 years, a percentage that grows every year^1^.

The "Big Five Factors" are basic dimensions of personality composed of facets that, according to their intensity, allow the identification of more likely patterns of behavior, beliefs and attitudes. In the evaluation of the 146 patients, there was a prevalence of the Neuroticism factor. About half of the respondents had high or very high scores for Neuroticism, indicating a more intense experience of emotional suffering, with a tendency to emphasize the negative aspects of events to the detriment of positive ones and even interpret ambiguous stimuli in a threatening way, as stated by Nunes et al.^15^. Among the high scores, females were identified as the majority. Most personality studies show that women score higher than men in Neuroticism, as revealed by Cheng et al.^16^, suggesting a greater tendency in females to emotional predominance at the expense of rationality.

It should be noted that Neuroticism is also associated with a greater propensity to develop depression and anxiety and is closely related to borderline, schizotypal, avoidant and dependent personality disorders^15^.

A study carried out by Bagnjuk et al.^17^ and Vainik et al.^18^ found a significant association between obesity and Neuroticism, as well as by Olivo et al.^19^, which pointed out, in addition to the association of the factor with overweight, also the worsening of cognitive performance. Since Neuroticism is the factor most associated with the individual's emotions and represents the chronic level of adjustment and instability in the face of psychological discomfort, we can suggest a greater tendency to eat in response to certain negative emotions, even motivating binge eating.

Looking at the most prevalent personality facets in Neuroticism, Depression was found in more than half of women and half of men. Low self-esteem, insecurity, difficulty in making decisions and dealing with daily challenges, negative expectation about the future, tendency to please others to the detriment of their own will, are likely patterns of behavior of these individuals.

Cheng et al.^16^ reveal the association between depression and obesity, both as a cause and as a consequence of obesity. They suggest that women tend to overeat as a kind of comfort when faced with stress and emotional problems, and excess weight, in turn, can lower self-esteem and worsen depression. Konttinen^20^ relates emotional eating - a tendency to higher intake of foods high in fat, sugar and salt - to atypical depression and obesity, understanding that negative emotions lead to emotional eating and the consequent development of obesity. Weight gain, in a cyclical way, can contribute to or aggravate depression, an issue observed mainly in women.

The bidirectional association between obesity and depression can be confirmed in this research, suggesting that women have greater difficulty in dealing with their negative emotions, due to high scores in Neuroticism, especially in the Depression facet, tending more to emotional eating and consequent obesity.

Neuroticism thus presented a significant relationship with obesity, highlighting the behavior patterns related to Depression, Vulnerability and Passivity. Most patients show a tendency to difficulty, both in dealing with daily adversities and in viewing the future from a positive perspective. These conducts could interfere in the preparation for bariatric surgery and in the process of recovery and change of post-surgical habits, especially care with food and physical activity.

The personality dimension classified as Extroversion is related to the individual's way of interacting with others and indicates the level of communication, speech, activity, assertiveness, responsiveness and community coexistence. High scores were found in just over a third of patients, who tend to be sociable, talkative, active, affectionate and optimistic. On the contrary, one-fifth of those surveyed had low scores and tended towards greater introversion, indifference, quietness and independence^15^.

The research found little significant difference when evaluating Extroversion in men and women, proportionally. In their study, Bagnjuk et al.^17^ found an association between obesity and Extroversion in both women and men. On the contrary, the study by Cheng et al.^15^ found a significant relationship in Extroversion and obesity only in males, suggesting greater social interaction among men in clubs and bars, leading to greater intake of drinks and food. In this study, we can suggest a relationship between obesity and Extroversion in both sexes, understanding that the social encounter in Brazilian culture is almost always mediated by food.

The most prevalent personality facet in the Extroversion dimension was that of haughtiness in women and Dynamism/assertiveness in men. High scores for the haughtiness facet suggest behaviors such as the need to receive attention from people, the belief that others envy them, and the predisposition to talk about oneself. High levels of Dynamism/assertiveness suggest behaviors of dynamism, involvement with several activities simultaneously and preference for keeping busy^15^.

The personality dimension classified as Socialization is related to the types of social interactions that the individuals present over time, their quality and how capable or compatible they feel in social life. High levels were found in just over a fifth of patients, indicating a tendency to trust people, be loyal and frank, and show a high level of altruism. They tend to be submissive and may spend more time and resources helping others. In contrast, people with low scores tend to distrust others, have few friends, and can be hostile and manipulative. They may also present high patterns of psychoactive substance use, disrespect for social rules and laws, and recurrent infidelity, characteristics associated with antisocial PD, as pointed out by Nunes et al.^15^.

In the survey, high levels of Socialization were found in a quarter of women and low levels in a third of men. A study by VanderBroek-Stice et al.^21^ relates impulsivity, obesity and compulsive eating pattern, pointing out behavioral and neurochemical parallels between psychoactive substance addicts and individuals with binge eating. Low levels of socialization in males may indicate a greater tendency to impulsivity, contributing to emotional eating, binge eating and possible obesity.

The Confidence in People personality facet that belongs to the Socialization dimension was more prominent, but at the lowest levels for this trait, corresponding to more than half of the women evaluated and just under half of the men evaluated. According to Nunes et al.^15^, low levels indicate a tendency to difficulty in developing intimacy with others, and may perceive them as dishonest, dangerous or causing some kind of damage. They are also associated with lower life satisfaction and positive affect. Based on the results found, we can associate low levels of Confidence in People with obesity. The individuals surveyed present greater life dissatisfaction and are more connected to negative affects, factors that could corroborate with emotional eating and possible post-bariatric weight regain.

The personality dimension classified as Achievement is characterized by organization, control, motivation and persistence in achieving objectives. High levels were found in almost a third of the patients, indicating, according to Nunes et al.^15^, a tendency to be more hardworking and dedicated towards their goals, even if this requires sacrifices.

Achievement is a personality factor associated with voluntary control, directing attention to certain actions, inhibiting, initiating or maintaining certain behaviors. About a quarter of the sample showed the opposite trend, giving up more easily in the face of difficulties and showing little motivation for complex tasks, more unpunctuality, disengagement and involvement in activities without clarity on how they will lead to their goals.

The results of the study may suggest that the patterns of effort and dedication behavior due to their own goals are protective factors in the postoperative period, taking into account the adaptations and new habits necessary for a good recovery and maintenance of weight loss. Low scores, notably present in females, reflect lower self-control and may be associated with difficulty in controlling food intake, contributing to obesity.

In the dimension of personality Achievement, the most prevalent facet in both women and men was Commitment, representing a predisposition to greater dedication to activities, perfectionism and detailed planning in the execution of tasks^15^. These results suggest that Commitment is a trait that favors commitment in the follow-up and completion of obesity treatment, involving preparation and post-surgery, both in men and women.

In addition, we evaluated the personality dimension classified as Openness, related to exploratory conduct and appreciation of new experiences. Low rates were found in most patients, indicating, according to Nunes et al.^15^, a tendency towards greater conservatism, dogmatism and rigidity, both in beliefs and attitudes and in preferences, in addition to less emotional responsiveness.

Vainik et al.^18^ found a correspondence between low Openness levels with less healthy diets and a greater tendency to obesity. Our study identified low Openness scores in most men, as well as in the group of women, a factor that can be associated with obesity, suggesting that these individuals have unhealthy eating behaviors and have greater difficulty with changing habits and routine.

The personality trait Openness to Ideas, belonging to the Openness dimension, presented higher scores for both women and men, at the lowest levels for this characteristic. It indicates a tendency, as presented by Nunes et al.^15^, to a rigid posture regarding concepts and preferences, in addition to little curiosity to know new themes and greater conservatism. These characteristics can be correlated with obesity, reinforcing habitual and repetitive eating patterns to the detriment of opening new experiences and understandings.

It is important to consider, according to Nunes et al.^15^ that mean scores are common to the general population and that very high or very low scores on certain factors and facets do not necessarily represent a maladaptive personality pattern. The results show trends in behaviors and probable patterns of attitudes and beliefs. The crossing of factors and/or facets may suggest the presence of PD and greater propensity to develop other psychiatric disorders.

Individuals with paranoid PD have high scores in Neuroticism, especially in the Emotional Instability facet, low scores in Socialization, with emphasis on Confidence in People and Social Interactions. High scores on Neuroticism and low scores on Achievement and the Confidence in People facet characterize borderline PD. In avoidant PD, there are high levels of Neuroticism and low levels of Extroversion and in the Competence facet. High scores on Haughtiness and low scores on Kindness and Pro-sociability are associated with narcissistic PD. In obsessive-compulsive PD, we found very high scores in Dynamism/assertiveness, high scores in Achievement and low scores in Openness, especially in Openness to ideas and Liberalism^15^.

The research results suggest the presence of personality disorders in 9% of the patients surveyed, confirming findings such as those of Mazer et al.^7^, who estimate the prevalence of PD between 9% and 15% in the adult population. Three patients met the criteria for paranoid PD, three for borderline PD, two for narcissistic PD, three for avoidant PD, and two for obsessive-compulsive PD.

There was a higher prevalence of PD belonging to groups B, characterized by emotionality and inconstancy, and of PD belonging to group C of the DSM-V, characterized by fear and anxiety. These are different findings from the study by Peluso et al.^22^, which found a predominance of group C.

We can suggest an association between the PD of groups B and C of the DSM-V with obesity, especially considering the high scores in Neuroticism common to both groups. However, despite the findings, it is important to note that no statistical tests were performed to identify possible correlations between the Big Five Factors and the selected variables, and further studies are needed to identify these associations.

Finally, significant differences were found between men and women in the Pro-Sociability of the “Socialization” factor and in the Search for News in the “Openness” factor in which a p value less than 0,05 was found. This can be explained by the fact that, first, the very nature of bariatric surgery and its physical and psychological consequences can affect men and women in different ways. In particular, bariatric surgery is associated with significant changes in lifestyle, body image, and social relationships, which may influence how patients perceive and interact with others^14^^,^^15^.

In addition, gender differences in social norms and cultural expectations can influence the way men and women express their sociability after surgery. For example, gender stereotypes can shape social interactions, leading men to exhibit sociability behaviors differently than women. Therefore, these differences may be reflected in the scores of personality facets, such as Pro-Sociability, resulting in significantly different p-values between gender groups^21^.

In addition, it is important to consider possible pre-existing differences in personality characteristics between men and women before bariatric surgery, which may influence how they respond and adapt to post-surgical changes. These individual differences may interact with the effects of surgery and contribute to the observed differences in Pro-Sociability between the sexes. Therefore, p-values less than 0.05 found can be attributed to a combination of factors, including the psychological consequences of bariatric surgery, gender norms, and individual differences in personality characteristics between men and women.

In this context, the role of nurses deserves to be highlighted, because when these professionals develop technical-scientific skills, they will be able to assist in the management of cases of bariatric patients in mental suffering, including personality disorder. The conduction may start with the application of tests, such as the BFP and nursing consultations aimed at listening to the biopsychosocial demands of the individuals and their family. With the identification of possible mental suffering, as in cases of PD, the nurses should be able to guide the patients and his/her family regarding the need to seek help or discuss the case with the multidisciplinary team, in addition to monitoring the case to verify nursing care needs, such as healthy lifestyle guidelines, diet, use of alcohol and other drugs, etc. The nurses stand out as important actors in the detection of suffering and guidance because they are professionals whose core is the care of human beings in all stages of life^22^.

The limitations of the study refer to the fact that the questionnaire was applied only in a bariatric surgery clinic that provides private care and by medical insurance, not reaching the population served by the Unified Health System (SUS). Another limitation refers to the research that occurred during the COVID-19 pandemic and may not have reached individuals who postponed surgery due to the risks of the pandemic.

## Conclusion

The extensive growth of obesity and the increasing search for bariatric surgery as a treatment for the disease raise the need for increasingly rigorous and cautious evaluations of these patients.

The dysfunctional patterns of behavior that stood out the most were related to the greatest difficulty in perceiving the positive side and ease in perceiving the negative side. There was also a predominance of dysfunctional patterns linked to rigidity of ideas and conduct. Half of the participants in this study presented high or very high scores for a greater propensity to develop depression and anxiety, showing a close relationship with personality disorders and the propensity to develop these conditions.

The results point to the need for a nursing team engaged with the purposes of assistance based not only on technical procedures, but also on the provision of psychological support and therapeutic listening, both in the preoperative period of bariatric surgery and in the postoperative period. Changing behavior patterns acquired since childhood requires long work but can contribute to greater emotional control and stimulate the change of physical and eating habits effectively.

More than that, the nursing team must be continuously trained and qualified with the multidisciplinary team to better identify dysfunctional behavioral patterns, focusing on patterns of effort and dedication in the completion of objectives, contributing to the success of the bariatric intervention.

The personality disorders identified in this study reinforce the importance of further research in the area that includes possible interventions to improve the psychic condition of these individuals, primarily before surgical intervention.
